# Gynecological and Obstetrical Society of Baltimore

**Published:** 1891-03

**Authors:** William S. Gardner

**Affiliations:** Secretary


### GYNECOLOGICAL AND OBSTETRICAL SOCIETY OF BALTIMORE.

Reported by WILLIAM S. GARDNER, M. D., Secretary.

### JANUARY MEETING.

THE president, Dr. HENRY M. WILSON, in the chair.

Dr. L. E. NEALE read a paper upon "*The Indications for Cesarean Section.*" This paper is intended to stimulate interest in and discussion of the subject Cesarean section vs. craniotomy on the living child, upon which subject a series of papers will be presented by the members of the Society. It refers particularly to the indications for the section, and is a plea for this operation.

If it serves to arouse interest in examining pelves, or increases hesitancy in destroying children, the labor is not in vain.

Craniotomy upon the living fetus is believed justifiable, but only as a dire necessity, not as an elective procedure, and should not be resorted to where there is a reasonable probability of success by the section, and the uncoerced consent of the mother can be obtained. No man is compelled to do craniotomy upon the living fetus solely upon the choice of the patient or her friends.

In answer to the question, "What would you do if the patient were your wife, your sister, or a near relative?" he believed practically this must be a matter for each man's conscience, over which no dogmatic rule of science can or should have sway.

If seen early enough, the induction of premature labor at the thirty-second or thirty-fourth week by the method of Krause was a very strong antagonist to craniotomy upon the living fetus. The range for this operation should not extend to a conjugata vera below $2\frac{3}{4}$ inches (7 cm.) or to one above $3\frac{1}{2}$ inches (8.75).

The indications for the conservative section included all insurmountable obstructions to the delivery of the living and viable child per *vias naturales.*

They include tumors, pelvic exudations, hypertrophic elongation of the cervix, cicatrices, stenoses, tetanus uteri, falciform uterine contractions, etc. He believed general opinion placed the limit for the absolute indication at a conjugata vera of $1\frac{1}{2}$ inches or 3.75 cm., and the relative indication extended from that point up to an undermined conjugata vera measurement, and included many other conditions besides pelvic contractions. Other things being factable, a $2\frac{1}{2}$ inch or 6.25 cm. conjugata vera (Harris) 3 inch, 7.5 cm. conjugata vera (Lusk) called for section rather than craniotomy, but be warned against relying *entirely* upon pelvimetry in the relative indications. In contracted pelves he preferred version to forceps when both were practicable. He insisted upon pelvimetry and briefly outlined the methods. He believed it was chiefly by this means we could determine the indication for the section.

A conjugata vera of 3 inches, 7.5 cm., was generally admitted to be the least through which a living child of normal proportions could pass, and as Lusk continued, if other diameters were lessened or the contraction was not limited to the brim, it might require a conjugata vera of 3½ inches, 8 cm. or more. No hard and fast line could be given; each case must be judged alone. The relative size of the head, its resistance, the past history, the uncoerced consent, the general condition and surroundings of the patient, etc., were all important factors in the relative indication.

The life of the child was not "purely impersonal and scientific," but eminently personal and practical, and he believed the mother should run a reasonable risk in its interest. The life-saving of craniotomy could never be as great as that of C. S., for it started with a necessary mortality of fifty per cent., or half the lives at stake. But aside from all argument and comparative statistics, the section was decidedly restricting craniotomy. All depreciated the repeated performance of craniotomy on the same woman. He accepted Carl Brann's rules for the relative indication.

Craniotomy was safer for the mother than section, but piecemeal extraction was equally if not more dangerous—ex. 92, conjugata vera 2½ inches 6.25 cm., or less.

If conservative delivery p. v. n. had been attempted and failed, this was a strong point in favor of craniotomy and against the section under these increased dangers.

He strongly deprecated conservative tampering, and then resorting to the section; many lives had been thus sacrificed. If we desired success we must make the section an elective operation, and not a procedure of dire necessity.

Dr. Miltenberger: With regard to the paper of Dr. Neale's, confined as it is to the indications for the Cesarean section, there is nothing which I would controvert. Under the absolute or positive indications as laid down, there can be no question. The confusion and discrepancy of opinion have arisen from want of definiteness and clearness as to the relative indications.

If we take the statistics of craniotomy generally, including all cases, we get no positive resulting data to guide us. Where the pelvis is so contracted as to necessitate the piecemeal extraction of the fetus, it is recognized, undoubtedly, as the most serious of obstetric operations, and more dangerous than Cesarian section.

Where, on the other hand, craniotomy alone is required, the operation is simple and the danger to the mother, in proper hands, should not be greater than from the application of the forceps. In my individual experience with my own patients, I have been obliged to resort to craniotomy but twice in fifty years, and in these, as well as in those in consultation practice, the mothers have all recovered.

Now it is just in this latter class that the doubt arises.

The smallest conjugata vera diameter through which a living child has been expelled is three inches, or, as has been claimed, $2\frac{3}{4}$ inches, but with this we cannot expect to save the child through the natural passages.

But whether with this or a little more available space, we must recognize the prime and absolute importance, as the doctor states, of pelvimetry, and to its thorough practical study and application must we mainly look for increased certainty. Especially does this hold as to external pelvimetry, the best instrument by far being the hand of the obstetrist.

Now, while it is true the measure here of the conjugata vera by the finger may not be perfectly accurate, and we require also to learn the available space in the transverse diameter, yet with care it sufficiently approximates the truth for our purpose.

But on the other hand, as the doctor has said, we cannot accurately determine the size of the child's head, its degree of ossification, etc. It is true by bimanual examination we can approximate the truth, but not exactly obtain it. I have known an accomplished accoucheur persist for a length of time in the use of forceps, before he recognized that he was dealing with a hydrocephalic head. Thus both the factors have elements of uncertainty. It is just in this class of cases that the doubt and uncertainty arises.

When the practical obstetrist meets with a case of dystocia, from this cause, by internal measurement he satisfies himself, as far as possible, he has three inches of available space in the conjugata vera, or even above this, without a full knowledge of the size of the fetal head, he naturally applies the forceps, or proceeds to turn and not improperly; but if he fails he has already violated the first fundamental law in Cesareotomy, to resort at first to the knife, without any previous operative manipulation, if such manipulation has been at all prolonged, the choice is not between craniotomy and Cesarean section, but between craniotomy and a Porro.

Fortunately, pelves contracted to this extent are rare in this country, particularly in the higher walks of life.

The operation of Cesareotomy is in itself sufficiently simple, and the modern section is, undoubtedly, one of the greatest advances in modern obstetricy, while its success constitutes a brilliant epoch in our recent history. In the hands of those skilled in the technique and taught and trained by experience, there is every reason to trust and believe that the modern Sänger will extend still farther its successes, and that as an operator gains tact and knowledge with every case with which he deals, and as a part of his success must depend upon his absolute command of his patient and her surroundings, it is most likely the old picture will be reversed, and with our septic and antiseptic precautions, hospitals will offer a smaller rate of mortality than private practice.

Fully realizing, as I do, the success of the modern Sänger, and the lessened mortality rate which has been achieved, yet we know that no abdominal section is entirely free from danger, and, as I said, in these cases of relative indication they may be claimed to be almost, if not entirely, void of peril with craniotomy.

I do not hesitate to declare that I should prefer, in my own wife, as the safer for her, craniotomy to Cesarean section in such a case, and am, therefore, bound to extend to others, my patients, the golden rule, "To do unto others, as I would they should do unto me." I am, therefore, forced to the opinion that Cesarean section will not completely supplant the old operation, and that there still remains a field, although markedly limited, for craniotomy on the living child.

Dr. J. Whitridge Williams: I am sure that all of us are greatly indebted to Dr. Neale for the very clear manner in which he has set forth the indications for the operation, and I almost entirely agree with him.

The absolute indication I would place at 5. 5½ cm., or three inches, and the upper limit for the relative indication at 7½ cm., or three inches. Within these limits, unless the child be abnormally small, there should be no question as to the use of forceps, and the question to be decided is whether craniotomy or Cesarean section should be done.

Theoretically, I would choose the section in all cases that appeared favorable, but practically, I might waive my theory in the case of a primipara who had not been examined previous to labor. For in that case it might appear very hard to submit a young woman to such a risk without any previous intimation of her danger.

But if I performed craniotomy under these circumstances, I would warn her that in becoming pregnant again she would take the responsibility of the child's life upon herself, and that I would refuse to perforate in subsequent pregnancies.

The mortality of the operation need not dismay us, for Munchmeyer has lately reported the latest statistics of Leopold, in which he reports twenty-eight Sänger operations, with the loss of three mothers and one child, and seven Porro operations with no maternal deaths.

Dr. B. B. Browne: I had a case recently upon which I did Cesarean section. The woman was twenty-seven years of age. She had had one child. Her labor was two years ago, when she had convulsions, and a craniotomy was done. As a result of injury received at this time, the vagina and uterus sloughed, and there was complete atresia of the vagina. This atresia was afterwards opened up, and she became pregnant.

The vagina was contracted by cicatricial bands, and an opening could be felt in the side of the cervix, but to the left of the opening was a cup-shaped cavity, which might have been the old cervix.

She was not sure of the time of impregnation. She was swollen and her urine solidified with albumin upon heating. Labor pains began December 20th, and continued for one or two days, but there was no dilatation. She came to the hospital December 22d. She had severe uterine contractions that day, and came for the purpose of having Cesarean section done. But next day the pains had all gone. The night of January 1st the waters broke, and severe pains began. The cicatricial bands about the cervix were cut, and Elliot's forceps were introduced. Both blades of Tarnier's forceps could not be gotten on. After several efforts I concluded that she could not be delivered in that way. In the morning the fetal heart was distinct, in the afternoon it was feeble.

The section was made without difficulty. The placenta was attached in front. The child could not be resuscitated. The placenta was readily detached and the uterus was cleaned out and closed by the Sänger method.

The operation was done on Friday, and the patient did well until the following Tuesday, when she sank rapidly, and died in a few hours. The woman had grave kidney disease, and had little chance of recovery on that account.

In this case several things are to be considered :

1st. The woman was perfectly willing for the operation.

2d. Her life, from the condition of her kidneys, was not insurable, and the child had a good chance of living.

3d. She had much difficulty in the former craniotomy; and barely escaped with her life.

Dr. Ashby : I have had the good fortune to witness two Cesarean sections. One, the case of Dr. J. G. Jay, of this city, several years ago, and the recent case reported by Dr. Browne. I was impressed with the case with which the operation can be done. Its mechanical execution is certainly much less difficult, than is necessitated by many intra-abdominal operations. Hemorrhage is easily controlled, and the closure of the uterine wound is not a difficult undertaking.

In the case of Dr. Jay, the mother made a prompt recovery, and the child perished simply because of the unavoidable delay which was experienced before an attempt at its removal was made. Its death had, in my opinion, no relation to the operation, but to causes which antedated the section. I am convinced in the case of Dr. Browne the child could have been saved had no other method of delivery been attempted.

Section, I think, bore no relation to its death. In this case the operation was skilfully done, and I am inclined to believe that the mother's death should be assigned chiefly to her kidney complications. She was a bad subject, but bore the section well. My opinion of Cesarean section is altogether favorable. It has come to stay, and with an improved technique and larger experience, will be approached with less hesitation.

The operation of the future will be approached without delay, and before other methods of delivery have been employed.

The important indication for the operation rests upon careful pelvic measurements and determination in advance of any obstetric interference of the improbability of delivery by version or forceps. If this is done, the section will be approached under its most favorable aspect and its results will be far more satisfactory.

I agree with Dr. Miltenberger in that personally I would prefer craniotomy, if the patient were a member of my own family, but upon scientific grounds, I would not hesitate to operate did my patient and her friends elect this procedure, having satisfied my own mind that a living child could not be born in any other way.

I think it unfortunate that the physician in charge of these cases should not have the moral support of the public and profession in the selection of the section in advance of attempts at other methods of delivery. Out of deference to a sentiment, he often feels forced to use the forceps and version where his own judgment was in favor of the section. Valuable time is thus lost, and the lives of both mother and child endangered, if not sacrificed.

Dr. NEALE: As no points were raised against the paper, I have nothing to say in its defense.

I did examine Dr. Browne's case, and told how, in my opinion, it was no case for the section. The chief obstruction was in the soft parts, that in the pelvis was very slight, if any. I thought it possible to deliver the child alive, p. v. n., but was sure it could be readily extracted after craniotomy. Owing to the kidney complication, the mother was in a most unfavorable condition for the section, and for that matter the child also; therefore, I advised against this operation.

However, after once beginning a conservative delivery, p. v. n., which was persisted in too long (thirty minutes), I certainly never should have resorted to the section in that case, with both child and mother in the then most unfavorable condition, but would have delivered at once by craniotomy.

I totally and emphatically differ from Dr. Ashby, that any conscientious obstetricians should ever be forced to resort to craniotomy by the moral evasion of the patient or her friends. Such teaching would be extremely pernicious.

The sentimental question of what one should do if the patient were his wife, etc., is a matter of individual conscience and not open to scientific discussion before a medical society.

I again request the fellows not to let this matter rest where we leave it tonight.

I wish to emphasize the fact that I have purposely avoided any reference to the religious aspect of this question, as I do not believe this work is open for scientific discussion before a medical society.

---

"IS THE doctor in?" asked a tramp at the door of an Arch street physician recently. A few minutes later an oldish female came to the door. "I just wanted to see if the doctor wouldn't give me a pair of his old pants," said the tramp. "I'm the doctor," said the lady. The tramp had several attacks of vertigo as he dropped down the steps.—*Boston Journal of Health.*

